# Supplementary material for: Innovative Approach to Isolate and Characterize Glioblastoma Circulating Tumor Cells and Correlation with Tumor Mutational Status
Source: Int J Mol Sci. 2023 Jun 14;24(12):10147. doi: 10.3390/ijms241210147 (PMC10299553; doi:10.3390/ijms241210147)
Supplement: Supplementary file 1 [file ijms-24-10147-s001.zip › ijms-2425210-supplementary.pdf]

Supplementary Data

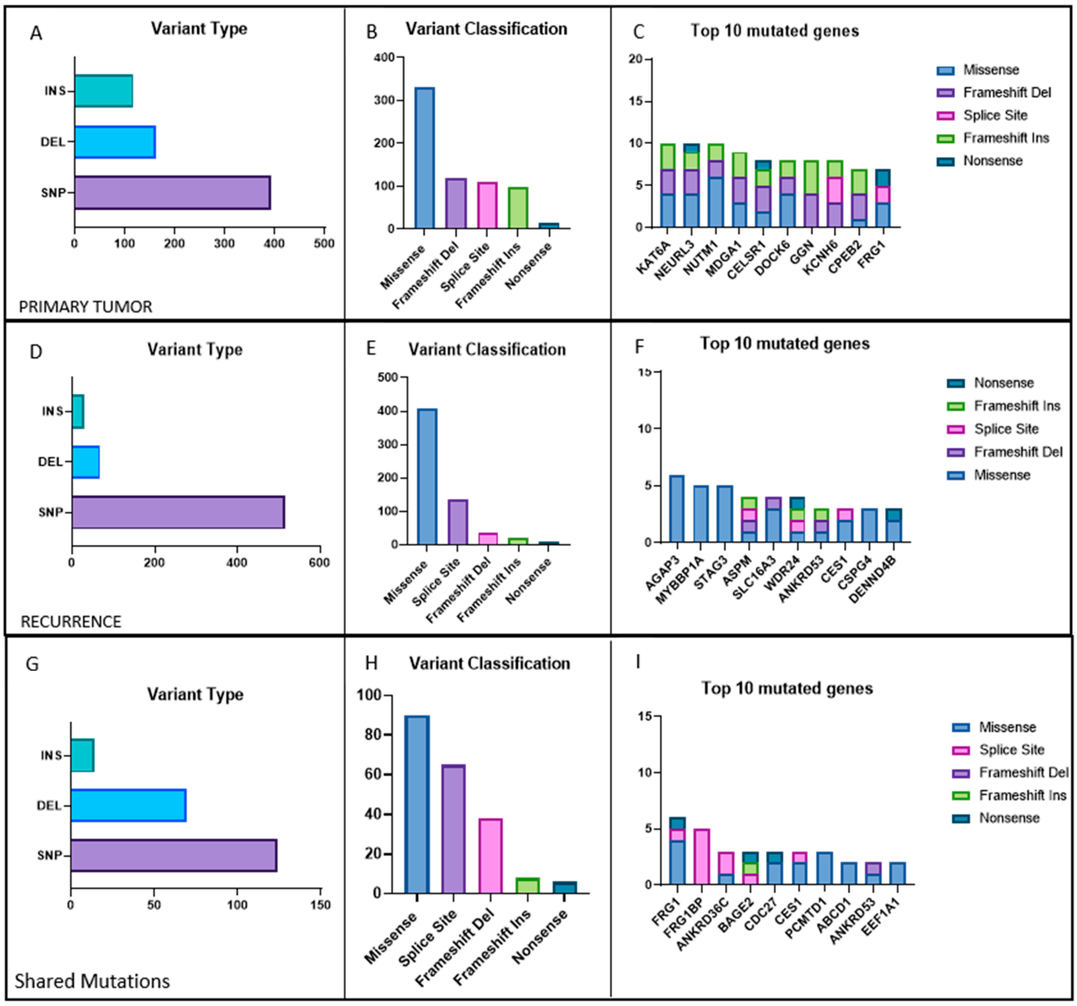

Figure 1S

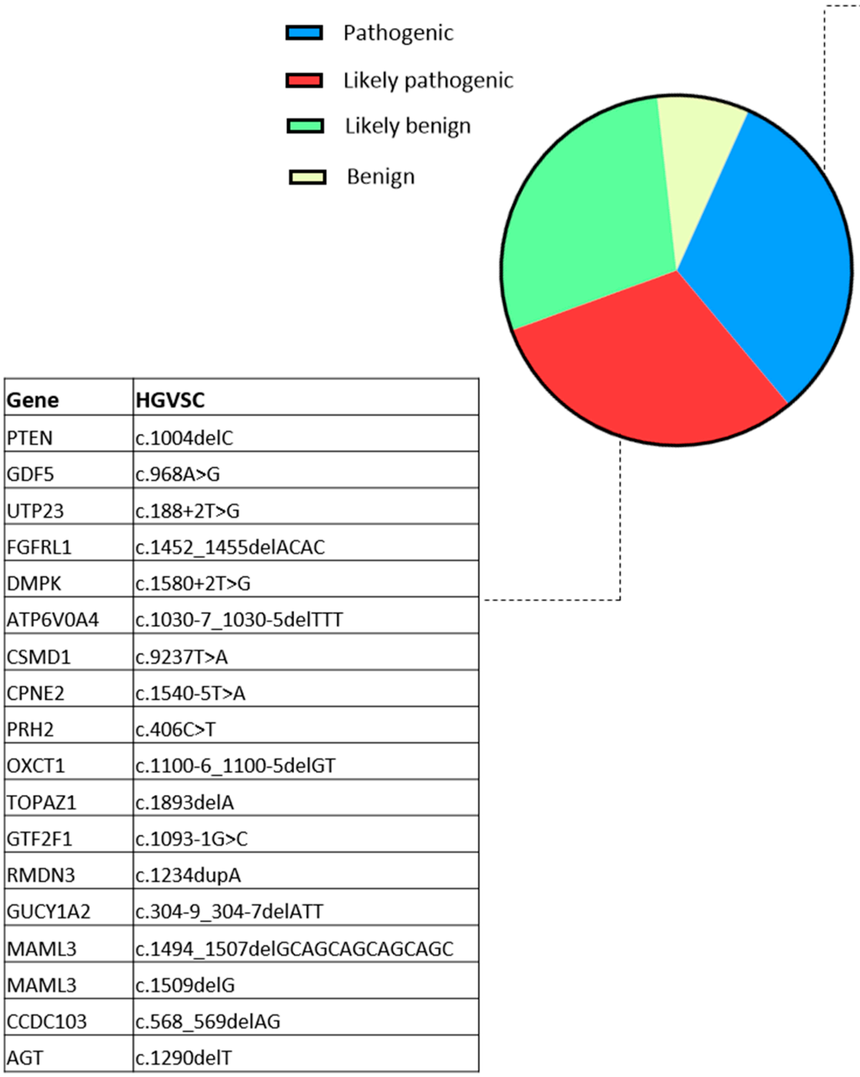

Gene	HGVSC
GLUL	c.476-1G>A
CREBBP	c.4478T>A
ACY3	c.664G>A
ANO8	c.2550+2T>G
FHOD3	c.1906_1907delAG
ABCD1	c.1823G>A
ABCD1	c.1816T>C
CEBPZ	c.1428delA
SBNO1	c.3416delA
CEP290	c.3175delA
MYO6	c.2751dupA
SMG7	c.2407dupA
LRCH1	c.483_486delCCTG
DDX27	c.2072delA
HNF1A	c.872dupC
SHC1	c.1087dupG
CISD3	c.186_187delTG
TNXB	c.703_704delTG
FANCM	c.2201_2202delCT

Gene	HGVSC
PTEN	c.1004delC
GDF5	c.968A>G
UTP23	c.188+2T>G
FGFRL1	c.1452_1455delACAC
DMPK	c.1580+2T>G
ATP6V0A4	c.1030-7_1030-5delTTT
CSMD1	c.9237T>A
CPNE2	c.1540-5T>A
PRH2	c.406C>T
OXCT1	c.1100-6_1100-5delGT
TOPAZ1	c.1893delA
GTF2F1	c.1093-1G>C
RMDN3	c.1234dupA
GUCY1A2	c.304-9_304-7delATT
MAML3	c.1494_1507delGCAGCAGCAGCAGC
MAML3	c.1509delG
CCDC103	c.568_569delAG
AGT	c.1290delT

Figure 2S

GBM Driver Genes from IntOGen	Primary tumor	Recurrent tumor	Concurrent Mutations
PTEN			X
NF1			
BRAF			X
AKAP9			X
ARID1A			
CAD			

SOX9*			X
MAP4K3			
WT1*			X
PBMR1			
CSDE1			
PRPF8			
CARM1			

Table 1S

Legenda Supplementary Data

Figure 1S

In Figure 1S, primary (A, B and C) and recurrent tumor (D, E and F) results are shown. In A, for the primary tumor the mutations are classified by type: 392 SNPs, 163 deletions and 117 insertions. While in B the variant classification plot shows the number and classification of the variants, in order of frequency. The same results are shown respectively for the recurrent tumor in D (mutations type plot) and E (mutations classification plot). Specifically, for the recurrent tumor the type of mutations are 515 SNPs, 68 deletions and 29 insertions. In C and F the top 10 mutated genes are reported together with mutations distribution for primary and recurrent tumor respectively. For primary tumor, the 10 most mutated genes are: KAT6A, NEURL3, NUTM1, MDGA1, CELSR1, DOCK6, GGN, KCNH6, CPEB2, and FRG1. For the recurrent tumor, the 10 most mutated genes are: AGAP3, MYBBP1A, STAG3, ASPM, SLC16A3, WDR24, ANKRD53, CES1, CSPG4 and DENND4B. In G and H the variant type and the classification plots are shown. In I the top 10 mutated genes in common between the tissues are reported: FRG1, FRG1BP, ANKRD36C, BAGE2, CDC27, CES1, PCMTD1, ABCD1, ANKRD53 and EEF1A1.

Figure 2S

Pie chart showing the 210 shared variants' classification. In blue the 18 classified as "pathogenic" are shown and are reported in the table above. In red the 19 the classified as "likely pathogenic" are shown and reported in the table below.

Table 1S

Analysis with IntOGene framework: from the Cancer Genome Atlas, 75 mutational cancer drivers were detected in the GBM project. 13/75 driver genes were present and distributed mainly in the primary tumor.

In particular*: NF1, BRAF, CAD, MAP4K3, PBMR1, CSDE1, PRPF8* mutated only in the primary tumor, *ARID1A and CARM1* were exclusively mutated in the recurrent tumor, and *PTEN, AKAP9, SOX9, WT1* and *BRAF* were altered in both the primary and recurrent tumors with the same mutation (except for *SOX9* and *WT1* with a different mutation). * For each gene the same mutations were present in primary and recurrent tumors except for SOX9 and WT1
